# Chicken IgY Fc Linked to *Bordetella avium* ompA and Taishan *Pinus massoniana* Pollen Polysaccharide Adjuvant Enhances Macrophage Function and Specific Immune Responses

**DOI:** 10.3389/fmicb.2016.01708

**Published:** 2016-11-01

**Authors:** Wenwen Dong, Hao Zhang, He Huang, Jianbo Zhou, Liping Hu, Ailing Lian, Lijun Zhu, Ningning Ma, Pingping Yang, Kai Wei, Ruiliang Zhu

**Affiliations:** ^1^Laboratory of Animal Biological Products, College of Animal Science and Technology, Shandong Agricultural UniversityTaian, China; ^2^Shandong New Hope Liuhe Co., Ltd, New Hope GroupQingdao, China; ^3^Animal Disease Prevention and Control Center of Shandong Province, Animal Husbandry and Veterinary Bureau of Shandong ProvinceJinan, China

**Keywords:** IgY Fc, *Pichia pastoris* expression, subunit vaccine, TPPPS, peritoneal macrophage

## Abstract

Fc-fusion technologies, in which immunoglobulin Fc is genetically fused to an antigenic protein, have been developed to confer antibody-like properties to proteins and peptides. Mammalian IgG Fc fusion exhibits improved antigen-induced immune responses by providing aggregates with high avidity for the IgG Fc receptor and salvaging the antigenic portion from endosomal degradation. However, whether the linked chicken IgY Fc fragment shares similar characteristics to mammalian IgG Fc remains unclear. In this study, we linked the chicken IgY Fc gene to the outer membrane protein A (ompA) of *Bordetella avium* through overlapping PCR. The fusion gene was cloned into the pPIC9 plasmid to construct the recombinant *Pichia pastoris* transformant expressing the ompA–Fc fusion protein. The effects of the linked Fc on macrophage vitality, activity, efficiency of antigen processing, and immune responses induced by the fused ompA were investigated. Furthermore, the effect of Taishan *Pinus massoniana* pollen polysaccharide (TPPPS), an immunomodulator, on chicken macrophage activation was evaluated. TPPPS was also used as an adjuvant to investigate its immunomodulatory effect on immunoresponses induced by the fused ompA–Fc in chickens. The pinocytosis, phagocytosis, secretion of nitric oxide and TNF-α, and MHC-II molecular expression of the macrophages treated with the fused ompA–Fc were significantly higher than those of the macrophages treated with ompA alone. The addition of TPPPS to the fused ompA–Fc further enhanced macrophage functions. The fused ompA–Fc elicited higher antigen-specific immune responses and protective efficacy compared with ompA alone. Moreover, the fused ompA–Fc conferred higher serum antibody titers, serum IL-2 and IL-4 concentrations, CD4+ and CD8+ T-lymphocyte counts, lymphocyte transformation rate, and protection rate compared with ompA alone. Notably, the prepared TPPPS adjuvant ompA–Fc vaccines induced high immune responses and protection rate. The linked Fc and TPPPS adjuvant can remarkably enhance macrophage functions and specific immune responses. This study provides new perspectives to improve the immune effects of subunit vaccines for prevention of poultry diseases.

## Introduction

Inactivated or killed vaccines have been widely applied to control infectious diseases. However, conventional formalin- or heat-inactivated vaccine formulations can alter the physiochemical/structural properties of the antigens, thereby negatively affecting the development of protective immunity (Jalava et al., 2002; Peng et al., 2011). Recombinant subunit vaccines can be used to effectively prevent bacterial diseases because of their resemblance to the native form as well as their rapid, consistent, and scalable production. Proteins or peptides generally show short serum half-life and limited antigenic stimulation because of conventional antigen capture by antigen-presenting cells (APC) and the fast renal clearance. Currently, nine human IgG1 fragment crystallizable (Fc) domain fusion drugs have been approved by the FDA to extend the serum half-lives of the linked antigens (Rath et al., 2015). Moreover, antigens synthetically linked to immunoglobulin IgG Fc molecules are more immunogenic than native antigens alone (Konduru et al., 2011; Zaharatos et al., 2011; Tayra et al., 2013; Iorio et al., 2015). Thus, the introduction of immunoglobulin Fc can definitely improve the immune effect of subunit vaccines.

Mammalian IgG can be divided into the Fab region, which binds to highly variable pathogenic antigens, and the Fc portion, which contains two constant domains on the C-terminal (Cγ2 and Cγ3 domains). IgG is involved in recruiting and activating immune effector leukocytes, such as macrophages, dendritic cells, and natural killer cells, thereby increasing the efficiency of these APCs for antigen elimination and presentation and triggering the functions of effector cells for the removal of infected cells (Jefferis, 2009). The fusion of immunoglobulin Fc to antigenic proteins confers aggregates with high avidity for the IgG Fc receptor (FcR) which widely exists in immune effector leukocytes (Nimmerjahn and Ravetch, 2008). Moreover, the Fc fusion leads to salvation of the antigenic portion from endosomal degradation by binding to the FcR of immunoeffector cells (Roopenian and Akilesh, 2007). Thus, Fc fusion technologies, in which immunoglobulin Fc is fused genetically to an antigenic protein, have been developed to confer antibody-like properties to proteins and peptides (Harrington et al., 2009). In avian species, the major immunoglobulin IgY is involved in humoral immunity against common avian pathogens. Although some studies have shown that IgY is similar to mammalian IgG in terms of functionality, the Fc segments between IgG and IgY exhibit different structures; the IgY Fc fragment contains two constant domains on the C-terminal (Cυ3 and Cυ4). A recent study has indicated that chicken IgY Fc expressed by *Eimeria mitis* enhances its immunogenicity (Qin et al., 2016). Thus, whether the linked chicken IgY Fc fragment fusion can also confer antigens more features to improve the antigen-induced immune response remains unclear.

Macrophages play a central role in immune defense mechanisms; these cells can not only initiate innate immune responses but are also involved in antigen processing and presentation to antigen-specific T cells to promote adaptive immunity. Both pattern recognition receptors and Fc–FcR interactions can activate macrophages *in vivo*. Fc–FcR interactions mediate antigen capture and influence the cytokine production of stimulated macrophages (Sutterwala et al., 1997). The engulfed antigens are digested into fragments and displayed on the cell surface by MHC-II molecules to present to T cells (Neild et al., 2005). However, whether the linked chicken IgY Fc fusion exhibits similar properties to natural Fc for macrophage activation are largely unknown.

Plant polysaccharides demonstrate immunoregulatory activity and potential irritation to macrophages (Jiang et al., 2010; Ma et al., 2010; Sun et al., 2015). Taishan *Pinus massoniana* pollen polysaccharide (TPPPS), a pleiotropic polysaccharide extracted from Taishan *P. massoniana* pollen, has been studied in our laboratory since 2003. TPPPS is an effective adjuvant for improving the immune system, facilitating immune responses, and enhancing the activity of lymphocytes (Wei et al., 2011; Cui et al., 2013; Yang et al., 2015). However, the influence of TPPPS on macrophage activity has not yet been studied.

In the present study, we used the *P. pastoris* GS115 eukaryotic expression system to express a fusion protein containing the chicken IgY Fc and ompA of *Bordetella avium*, a common respiratory disease pathogen of avian species (Gentry-Weeks et al., 1992). The influence of IgY Fc fusion on immune responses induced by the ompA antigen was then evaluated. TPPPS was also used as an immune adjuvant to investigate its irritation on chicken peritoneal macrophages and immunomodulation on ompA–Fc-induced immunoresponses. This study mainly aims to explore a feasible method for improving the immune effects of subunit vaccines for poultry.

## Materials and Methods

### Ethics Statement

The animal disposal procedures were approved by the Animal Care and Use Committee of Shandong Agricultural University (Permit no. 20010510) and performed according to the “Guidelines for Experimental Animals” of the Ministry of Science and Technology (Beijing, China).

### Strains and Plasmids

*Bordetella avium* strain LL was isolated from sick chickens and preserved in our laboratory. The genetic homology of 16S rRNA between *B. avium* strain LL and the reference strain S5 was 100%. The strain was cultured and maintained at 37°C in lysogeny broth agar. *P. pastoris* GS115 and plasmid pPIC9 were purchased from Invitrogen (Carlsbad, CA, USA). The recombinant pPIC9-ompA plasmid was provided by our laboratory. All yeast culture media were prepared in accordance with the manuals of *Pichia* expression.

### Expression and Identification of the Recombinant ompA–Fc

One pair of primers (F1: 5′-CCGCTCGAGATGCATCATCATCATCATCATAACAAACCCTCCAAAATCGCACTT-3′; R1: 5′-CGTATGAACCTCCACCTCCTGATCCACCTCCACCCTTGCGGCTACCGACGATTT-3′) was designed according to the ompA gene sequence of *B. avium* (GenBank accession number: M96550.1) by using Primer 5.0. Another pair of primers (F2: 5′-CAAGGGTGGAGGTGGATCAGGAGGTGGAGGTTCATACGCCATCCCACCCAG-3′; R2: 5′-TTGCGGCCGCTTAATGATGATGATGATGATGGCGCTGGCTGAAGCGGATG-3′) was designed to produce hinge–C_H_3–C_H_4 (Fc), a 692 bp fragment Fc-linker, by using Primer 5.0 software according to the IgY Fc gene sequence in chickens (GenBank accession number: X07174). The underlined bases encode flexible linker peptides. The target genes were assembled through overlapping PCR. One pair of primers (F1 and R2) was designed to amplify a 1293 bp fragment ompA-linker-Fc. The linked ompA–Fc was cloned into the expression vector pPIC9 and named pPIC9-ompA–Fc. The resultant plasmid was confirmed by sequencing (Sunny, Shanghai). The plasmid was then transformed into competent *P. pastoris* GS115 to obtain the transformant *P. pastoris* pPIC9-ompA–Fc in accordance with the manufacturer’s instructions (Invitrogen). The recombinant protein ompA–Fc was identified using SDS-PAGE. Western blot analysis was performed using mouse anti-omp polyclonal antibody [prepared in accordance with our previous method (Hu et al., 2007)], anti-His tag antibody (Cwbio, China), and rabbit anti-chicken IgG (HRP) (Solarbio, China) (Temple et al., 2010). The recombinant pPIC9-ompA plasmid served as normal control and then transformed into *P. pastoris*. The recombinant ompA and ompA–Fc were purified by ProteinIso^TM^ Ni-NTA Resin kit (TRANS, Beijing, China). Protein concentration was determined by Easy II Protein Quantitative Kit (BCA) (TRANS, Beijing, China).

### Macrophage Cell Activity Assay

Chicken peritoneal macrophages were isolated as previously described (Mahapatra et al., 2009). The macrophages were cultured into 96-well cell culture plates and then divided into three groups. Then, ompA–Fc (2.5, 5, and 10 μg/mL), ompA (2.5, 5, and 10 μg/mL), and TPPPS (12.5, 25, 50, and 100 μg/mL) were separately added into the cells. After 24 h incubation, the vitality of peritoneal macrophages was determined by MTT assay (Mosmann, 1983). The neutral red uptake, nitric oxide (NO), and TNF-α production of peritoneal macrophages were detected as previously described (Ding et al., 1988; Sun et al., 2015).

The phagocytic activity of peritoneal macrophages was further determined. In brief, 5 μg/mL ompA–Fc mixed with 50 μg/mL TPPPS, 5 μg/mL ompA–Fc, 5 μg/mL ompA, and 50 μg/mL TPPPS and PBS was prepared. A 100 μL aliquot of each solution was added into 96-well cell plates for the culture of peritoneal macrophages. After 6 h of incubation, immunofluorescent assay (IFA) was performed to analyze ompA phagocytosis. Rat anti-omp polyclonal antibody was used as primary antibody, and FITC-conjugated mouse anti-rat IgG (Sigma, China) was used as secondary antibody. The plates were incubated one after another.

MHC-II molecules of macrophages were also determined. In brief, 5 μg/mL ompA–Fc mixed with 50 μg/mL TPPPS, 5 μg/mL ompA–Fc, 5 μg/mL ompA, and 50 μg/mL TPPPS and PBS was prepared. A 400 μL aliquot of each solution was added into 24-well cell plates for the culture of peritoneal macrophages. After 6 h of incubation, the cells were incubated with mouse anti-chicken MHC-II antibody (Abcam, China) for 30 min and then stained with FITC-conjugated goat anti-mouse antibody (Cwbio, China) for 30 min. The expression of MHC-II molecules on the surface of macrophages was detected by flow cytometry analysis (Guaga Easy Cyte Mini, USA).

### Vaccine Preparation

Taishan *Pinus massoniana* pollen polysaccharide was prepared by our laboratory through hot water extraction and ethanol precipitation (Wei et al., 2011). The purified recombinant ompA and ompA–Fc were diluted to final concentrations of 100 and 200 μg/mL, respectively, to ensure equal antigen content of ompA. TPPPS was mixed with 200 μg/mL purified ompA–Fc fusion protein at a ratio of 1:1 to a final concentration of 50 mg/mL. Stability and sterility tests were performed using the prepared subunit vaccines.

### Animal Experiment

A total of 120 1-day-old specific pathogen-free white leghorn chickens (male; Spirax Ferrer Poultry Co., Ltd, Jinan) were randomly placed into four sterilized isolators (groups I–IV), with 30 chickens each. Chickens in groups I–IV were subcutaneously inoculated with 0.2 mL of recombinant ompA vaccines, ompA–Fc fusion protein vaccine, TPPPS adjuvant ompA–Fc fusion protein vaccines, and PBS at 1, 7, and 14 days post first vaccination (dpv). Groups I, II, III, and IV were labeled ompA, ompA–Fc, ompA–Fc-TPPPS, and PBS, respectively. At 3, 7, 14, 21, 28, 35, 42, and 49 dpv, three chickens from each group were selected randomly to determine the levels of antibody, serum IL-2, and IL-4, CD4+ and CD8+ T lymphocyte counts in peripheral blood, and T-lymphocyte transformation rate. The chickens were not fed for 12 h before sampling.

At 1 week after the third vaccination (21 dpv), 20 chickens from each group were placed in a new isolator and challenged intranasally with 10 median lethal doses (LD_50_) of the *B. avium* LL strain. Clinical manifestation of the chickens was recorded for 7 days after the challenge. Clinical symptoms, including labored breathing, sneezing, and oculonasal discharges, were monitored (Saif et al., 1981). Three independent experiments were conducted, and the mortality and protection rate in each group were calculated using the following formulas:

$$\begin{array}{c} \text{Morbidity}\;(\%)=\frac{\text{No}.\;\text{of}\;\text{chickens}\;\text{with}\;\text{clinical}\;\text{symptoms}}{\text{Total}\;\text{No}.\;\times \;\text{100}.}\\ \text{Protective}\;\text{rate}\;(\%)=\frac{\text{No}.\;\text{of}\;\text{chickens}\;\text{without}\;\text{clinical}\;\text{symptoms}}{\text{Total}\;\text{No}.\;\times \;\text{100}.} \end{array}$$

### Detection of Immune Indices

Three blood samples (1.0 mL/chicken) from each group were randomly sampled at specific times. Indirect enzyme-linked immunosorbent assay (ELISA) was performed to detect anti-ompA antibodies (Denac et al., 1997). Cytokines are crucial in fighting infections and are involved in immune responses (Lowry, 1993). IL-2 and IL-4 were detected using their corresponding ELISA kits for chicken (Langdon Bio-technology Co., Ltd., Shanghai). Absorbance was determined with a microplate reader at 450 nm.

Fresh anticoagulated (EDTA–Na_2_) peripheral blood samples were randomly collected from three chickens (1.0 mL/chicken) in each group and separately mixed with an equivalent volume of PBS. In brief, 2 mL of the mixture was added to 5 mL of lymphocyte separation medium (Solarbio, China) to separate lymphocytes (Mwanza et al., 2009). The percentages of CD4+ and CD8+ T lymphocytes were detected by flow cytometry (Guaga Easy Cyte Mini, USA). Lymphocyte proliferation was determined by MTT assay. In brief, the peripheral blood lymphocytes were isolated from the vaccinated animals, and the cultured lymphocytes were re-stimulated with ompA unfused to IgY. Then, the lymphocyte proliferation assay was performed as previously described (Mosmann, 1983).

### Statistical Analysis

Data were expressed as mean ± standard deviation (SD). Duncan’s multiple-range test was performed to analyze differences among groups by using SPSS 17.0. Values at *P* < 0.05 were considered statistically significant.

## Results

### Expression and Identification of the Fused ompA–Fc

**FIGURE 1:**
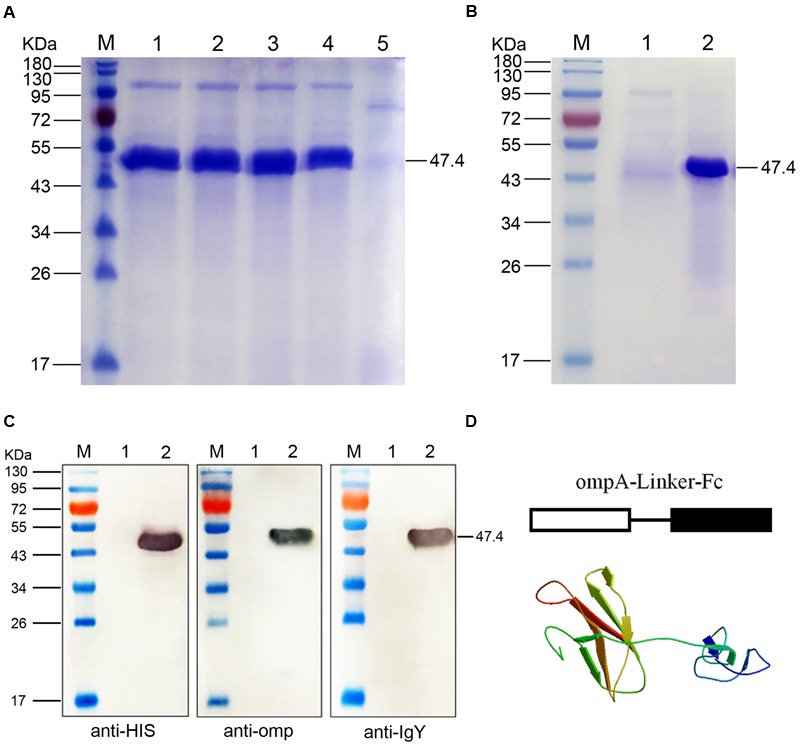
**SDS-PAGE and Western blot analyses of the fused ompA–Fc expressed in *Pichia pastoris*. (A)** SDS-PAGE identification of the fusion ompA–Fc at different induction times. M, Page ruler pre-stained protein ladder; lanes 1–4, culture supernatant of *P. pastoris* transformed with the recombinant pPIC9-ompA–Fc plasmid after 96, 72, 48, and 24 h of methanol induction; lane 5, culture supernatant of *P. pastoris* transformed with blank pPIC9 vector (negative control). **(B)** Purification of the fused ompA–Fc. M, Page ruler pre-stained protein ladder; lane 1, purified ompA–Fc; lane 2, culture supernatant after column chromatography. **(C)** Western blot analyses of the fused ompA–Fc with the anti-His tag antibody, mouse anti-omp polyclonal antibody, and the rabbit anti-chicken IgG (HRP). M, protein molecular size page ruler; lane 1, culture supernatant of *P. pastoris* transformed with blank pPIC9 vector (negative control); lane 2, culture supernatant of *P. pastoris* transformed with the recombinant pPIC9-ompA–Fc plasmid at 96 h post induction. **(D)** Schematic and 3D structure of the fused ompA–Fc.

The chicken IgY Fc and *B. avium* ompA genes were linked through overlapping PCR. The linked ompA–Fc gene fragment was cloned into the expression vector pPIC9 and verified by gene sequencing. The recombinant pPIC9-ompA–Fc plasmid was then transformed into *P. pastoris*. Upon induction with methanol at different induction times, a novel protein band corresponding to 47.4 kDa in the culture supernatant of the recombinant pPIC9-ompA–Fc transformant was determined through SDS-PAGE; however, this band did not appear in the culture supernatant of the control pPIC9 (blank plasmid) transformant (**Figure 1A**). The protein was detected in the supernatant after 48 h of cultivation, and the maximum protein yield (18 mg/L) was obtained at 72 h. After purification, only the target protein band with a molecular weight of 47.4 kDa was observed in the results of SDS-PAGE (**Figure 1B**).

Western blot analyses were performed with anti-His tag antibody, mouse anti-omp polyclonal antibody, or rabbit anti-chicken IgG (HRP) to determine the immunogenicity of the fusion protein. After color development, a 47.4 kDa band was observed in the three independent assays (**Figure 1C**); this band corresponded to the band detected in SDS-PAGE. The results indicated the independent immunogenicity of the fused IgY Fc and ompA of *B. avium*. Moreover, at the start of ompA–Fc fusion, antigens expressed in *P. pastoris* were presented in schematic and 3D structures of the fusion protein ompA–Fc and developed through homology modeling methods of SWISS-MODEL (**Figure 1D**). In addition, the recombinant pPIC9-ompA plasmid constructed in our laboratory was used as control in the present study; the immunogenicity of the recombinant ompA expressed in *P. pastoris* was also verified (Liu et al., 2016).

### Influences of the Linked Fc and TPPPS on the Viability of Peritoneal Macrophages

**FIGURE 2:**
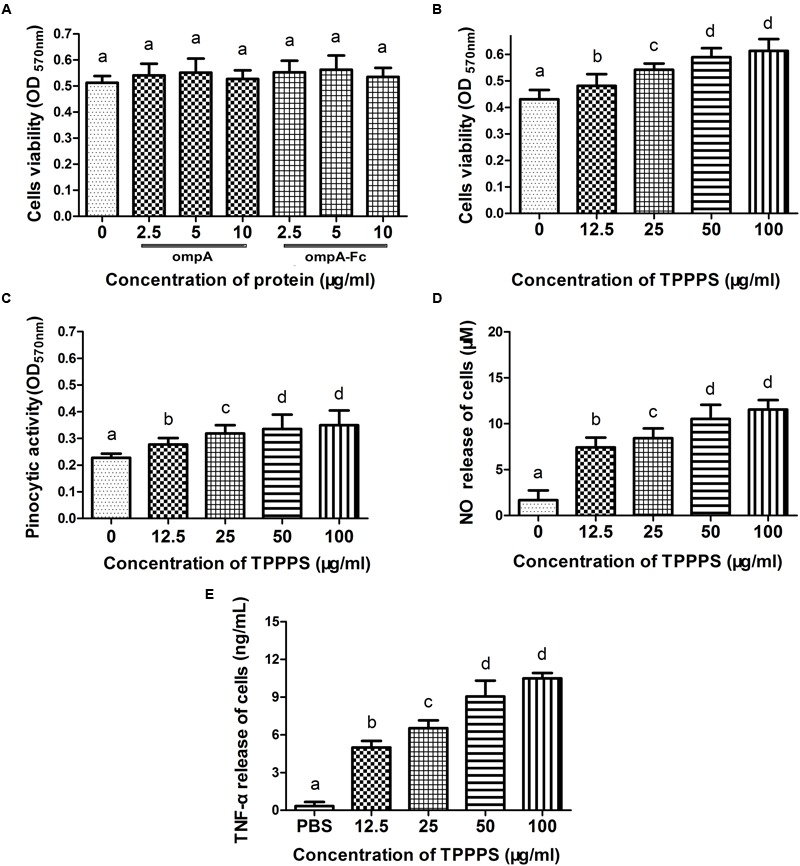
**Influences of the linked Fc or Taishan *Pinus massoniana* pollen polysaccharide (TPPPS) on macrophage activity.** Peritoneal macrophages were cultured with the various concentrations of ompA–Fc (2.5, 5, and 10 μg/mL), ompA (2.5, 5, and 10 μg/mL), TPPPS (12.5, 25, 50, and 100 μg/mL), and PBS for 24 h. The activity of peritoneal macrophages was determined by MTT assay **(A,B)**. The neutral red uptake **(C)**, NO **(D)**, and TNF-α **(E)** production of peritoneal macrophages were detected as previously described. All values represent the means ± SD of triplicate experiments. Different lowercase letters above the columns indicate significant differences between the different groups (*P* < 0.05).

The viability of macrophages treated with the recombinant ompA and ompA–Fc was assayed by MTT to assess the effects of the recombinant proteins on the growth of chicken peritoneal macrophages. The cytotoxicity of TPPPS, which was used as the adjuvant in the following experiments, was examined. Cell viability in the groups treated with the recombinant ompA (2.5–10 μg/mL) and ompA–Fc (2.5–10 μg/mL) was not significantly different from that in the control group (*P* > 0.05; **Figure 2A**). However, treatment with 12.5–100 μg/mL TPPPS increased cell viability compared with those in the control group (*P* < 0.05; **Figure 2B**). Cell viability improved with increasing concentration of TPPPS. The results indicated that treatment with recombinant proteins did not change the viability of chicken peritoneal macrophages, whereas TPPPS dose dependently promoted the proliferation of peritoneal macrophages.

### TPPPS Increases Pinocytic Activity, NO Level, and TNF-α Release of Peritoneal Macrophages

Internalization in the form of pinocytosis or phagocytosis is a key indicator of macrophage effector activity. NO and TNF-α secreted by macrophages also reflect cell activity. The effect of TPPPS on the pinocytic activity of peritoneal macrophages was assayed via neutral red uptake. The peritoneal macrophages treated with TPPPS (12.5–100 μg/mL) showed higher absorption values than those treated with PBS (*P* < 0.05; **Figure 2C**). Moreover, limited NO was released from the peritoneal macrophages in the PBS group, whereas NO production significantly enhanced as the amount of TPPPS added was increased (*P* < 0.05; **Figure 2D**). The level of TNF-α secreted by the TPPPS-treated macrophages was also significantly higher than that in the PBS group (*P* < 0.05; **Figure 2E**). The three indices were dose dependently improved by TPPPS but were not significantly different between the groups treated with 50 and 100 μg/mL TPPPS. Thus, we selected 50 μg/mL TPPPS as the adjuvant for the subunit vaccine.

### Linked Fc and TPPPS Adjuvant Enhance the Antigen Procession of Macrophages

**FIGURE 3:**
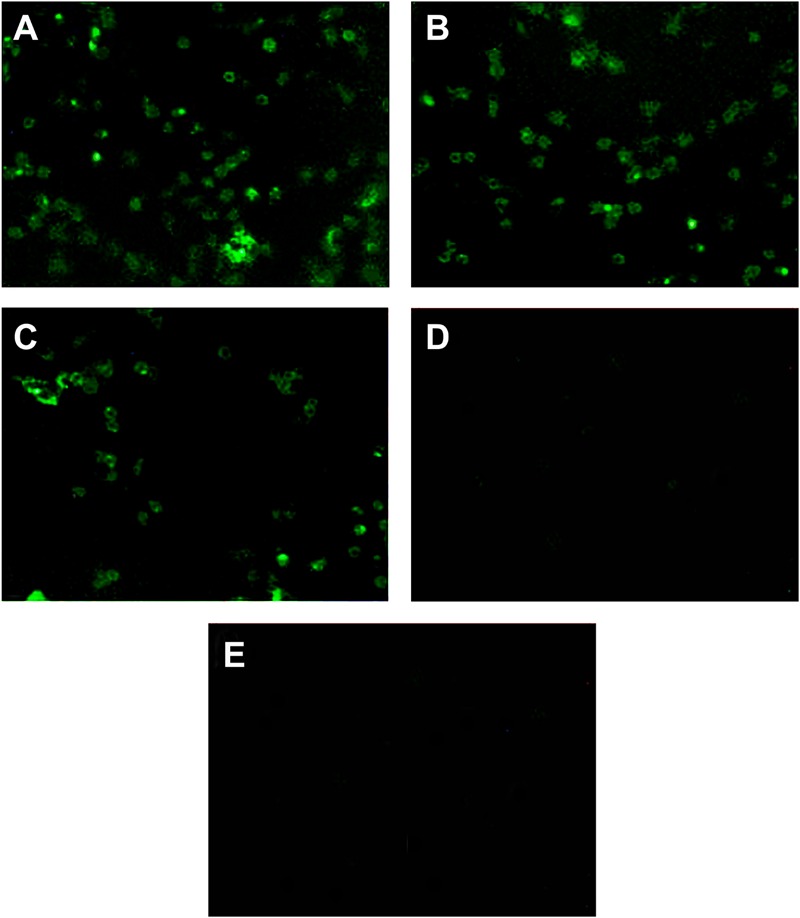
**The linked Fc and TPPPS adjuvant enhance phagocytosis of peritoneal macrophages.** Initially, 5 μg/mL ompA–Fc mixed with 50 μg/mL TPPPS **(A)**, 5 μg/mL ompA–Fc **(B)**, 5 μg/mL ompA **(C)**, and 50 μg/mL TPPPS **(D)** and 100 μL PBS **(E)** were separately added into 96-well cell culture plates, where fresh peritoneal macrophages were cultured. After 6 h incubation, IFA was used to analyze the ompA phagocytosis, and the rat anti-omp polyclonal antibody as primary antibody and FITC-conjugated mouse anti-rat IgG as secondary antibody were incubated successively.

**FIGURE 4:**
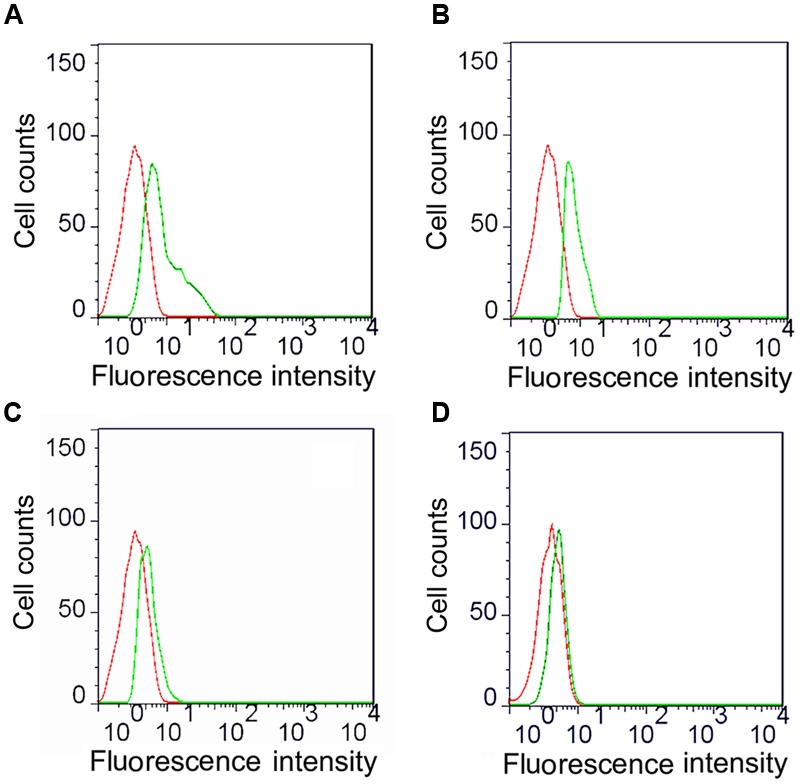
**Flow cytometric analysis of MHC-II molecules expressed on macrophages.** The macrophages were cultured into 24-well cell culture plates and 5 μg/mL ompA–Fc mixed with 50 μg/mL TPPPS **(A)**, 5 μg/mL ompA–Fc **(B)**, 5 μg/mL ompA **(C)**, and 50 μg/mL TPPPS, **(D)** and 400 μL PBS were added into each well. After 6 h incubation, cells were incubated with 10 μL of mouse anti-chicken MHC-II antibody for 30 min and then stained with 1:500 FITC-conjugated goat anti-mouse antibody for 30 min. The expression of MHC-II molecules on the surface of macrophages was detected by flow cytometry. Red dotted line: PBS-treated macrophages (control group); green dotted line: recombinant protein and/or TPPPS-treated macrophages (experimental group). The images are representative of three independent experiments.

We analyzed the ompA phagocytic efficiency and MHC-II expression of chicken peritoneal macrophages through IFA and flow cytometry, respectively, to detect the effects of the linked Fc and TPPPS adjuvant on the antigen procession efficiency of macrophages. Green fluorescence was observed on the ompA- and ompA–Fc-treated peritoneal macrophages (**Figures 3A–C**), but no fluorescence was detected on the PBS- and TPPPS-treated groups (**Figures 3D,E**); this finding indicated the phagocytosis of macrophages on the recombinant proteins. The staining density significantly enhanced in group ompA–Fc compared with that in group ompA (*P* < 0.05; **Figures 3B,C**). Moreover, the addition of TPPPS to ompA–Fc enhanced the fluorescence staining on the cells (**Figure 3A**). Linked Fc and TPPPS adjuvant remarkably increased the antigen capture capacity of the chicken peritoneal macrophages. Similarly, MHC-II expression was higher in the macrophages treated with ompA than in the macrophages treated with PBS and TPPPS (*P* < 0.05; **Figures 4C,D**); by contrast, the macrophages treated with the fused ompA–Fc showed higher MHC-II expression (*P* < 0.05; **Figure 4B**). The addition of TPPPS further enhanced MHC-II expression on the fused ompA–Fc-treated macrophages (*P* < 0.05; **Figure 4A**).

### Linked Fc and TPPPS Adjuvant Enhance Humoral and Cell-Mediated Immune Responses

**FIGURE 5:**
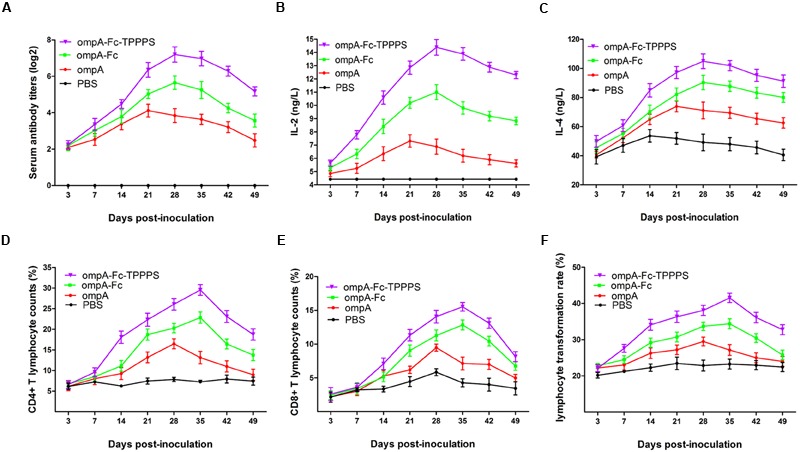
**Linked Fc and TPPPS adjuvant enhance the humoral and cell-mediated immune responses.** Chickens in the four groups were, respectively, inoculated with ompA subunit vaccine, ompA–Fc subunit vaccine, TPPPS adjuvant ompA–Fc subunit vaccine, and PBS at 0, 7, and 14 dpv. The specific anti-ompA antibody titers **(A)** were determined by indirect ELISA as previously described, and the concentrations of IL-2 **(B)** and IL-4 **(C)** were determined by commercial ELISA kits; the percentages of CD4+ **(D)** and CD8+ **(E)** T lymphocytes and LTRs **(F)** were detected by flow cytometry and MTT assay, respectively. All values represent the means ± SD of three independent experiments.

Antibody levels induced by vaccination are crucial in the determination of the effects of vaccines. Evaluation of antibody induction showed that the anti-ompA IgG titers in chickens vaccinated with the recombinant ompA were significantly higher than those in the control PBS group at 14–49 dpi (*P* < 0.05; **Figure 5A**). Anti-ompA IgG response was significantly higher in group ompA–Fc than in group ompA at 21–49 dpi (*P* < 0.05). Moreover, TPPPS as the adjuvant significantly promoted the antibody titers than ompA–Fc at 21–49 dpi (*P* < 0.05). In addition, the IgG titer in group ompA peaked at 21 dpv, whereas those in groups ompA–Fc and ompA–Fc-TPPPS peaked at 28 dpv.

Cytokines IL-2 and IL-4 in serum were measured to characterize cellular immune responses. The number and proliferative capability of T lymphocytes are common indicators used to evaluate cellular immunity (Torti et al., 2012). In the present study, the serum IL-2 and IL-4 concentrations in group ompA were significantly higher than those in group PBS at 14–42 dpv (*P* < 0.05; **Figures 5B,C**). These indices were significantly enhanced in group ompA–Fc than in group ompA at 21–42 dpv (*P* < 0.05). Moreover, the addition of TPPPS increased the serum IL-2 and IL-4 concentrations (*P* < 0.05). A similar trend was observed in the results of the relative populations of CD4+ and CD8+ lymphocytes in peripheral blood and lymphocyte proliferation abilities (**Figures 5D–F**). Linked Fc and TPPPS adjuvant effectively improved humoral and cellular immune responses induced by the ompA antigen in chickens.

### Linked Fc and TPPPS Adjuvant Enhance the Protective Effects Induced by the Recombinant ompA in Chickens

**FIGURE 6:**
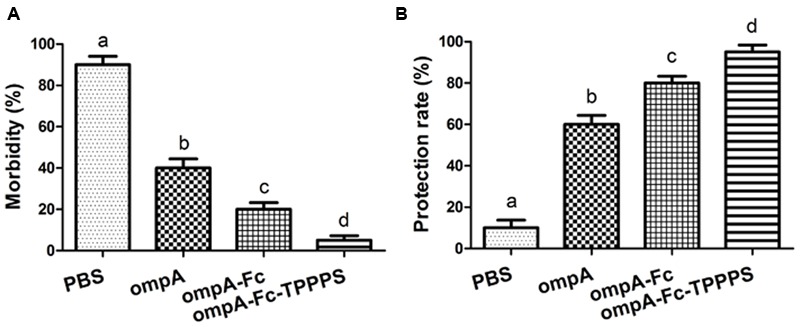
**Protective rates of *Bordetella avium*-challenged chickens.** Chickens in four groups were inoculated with ompA subunit vaccine, ompA–Fc subunit vaccine, TPPPS adjuvant ompA–Fc vaccine, and PBS at 1, 7, and 14 dpv. One week after the third vaccination, 20 chickens from each group were challenged intranasally with 10 LD_50_ of *B. avium* LL strain. Morbidity **(A)** and protective rate **(B)** were monitored for seven successive days after challenge. Morbidity (%) = No. of chickens with clinical symptoms/Total No. × 100. **(B)** Protective rate (%) = No. of chickens without clinical symptoms/Total No. × 100. Values shown represent the means ± SD for three independent experiments. Different lowercase letters above the columns indicate significant differences between the different groups (*P* ≤ 0.05).

Chickens in the four groups were challenged intranasally with 10 LD_50_ of *B. avium* LL strain at 21 dpv to evaluate the protective effects of the prepared vaccines. The clinical symptoms and protective rate of the chickens were monitored daily. After 7 days of observation, 93.3% of chickens in the control group showed symptoms, such as labored breathing, sneezing, and oculonasal discharge, after *B. avium* challenge (**Figure 6A**). By contrast, the morbidities in groups ompA–Fc-TPPPS, ompA–Fc, and ompA were approximately 6, 20, and 40%, respectively. The immunogenicity of the fused ompA–Fc subunit vaccine could remarkably protect chickens against *B. avium* infection, and the TPPPS adjuvant conferred optimal protection (**Figure 6B**).

## Discussion

Antibody Fc region is a recruiter and a frontline commander to protect against cancer and infectious pathogens by mediating potent immune effector functions by engaging FcR and serum complement proteins. Investigators sought to improve vaccine immunogenicity by incorporating entire proteins, discrete B-cell, or MHC classes I or II epitopes within IgG Fc scaffolds (Brumeanu et al., 1993; Cook and Barber, 1995; Lu et al., 2011). In the present study, we linked the chicken IgY Fc gene to the *B. avium* ompA gene to express the coded fusion protein in *P. pastoris*. Accordingly, we synthetically joined IgY Fc via the flexible hinge region to *B. avium* ompA to preserve the independent space structure of the fused sections. We predicted that the effector functions of the native IgY Fc would be retained because the C_H_3–C_H_4 regions of IgY Fc contain all critical residues necessary for interaction with effector cells. After verification, we found that the linked chicken IgY Fc improved the macrophage antigen processing efficiency and enhanced the immune responses induced by the ompA antigen.

The fused ompA–Fc protein was expressed in the *P. pastoris* expression system; this eukaryotic expression system exhibits advantages, such as easy cultivation, high yield, precise post-translational modifications, and minimal interference of native proteins (Cereghino and Cregg, 2000). This secretory expression system also facilitates subsequent purification, thereby avoiding damage to the content and activity of the recombinant ompA–Fc. In addition, the yeast-expressed antigen has been investigated in several animals and proven to exert satisfactory effects on the development of bacterial vaccines (Amigorena and Bonnerot, 1999). Basing on our results, we observed that the protein was detected after 48 h and reached the peak after 72 h of induction with 18 mg/L methanol. This foreign protein was secreted at high amounts in the medium. By contrast, the amount of native proteins of *P. pastoris* secreted in the medium was low, which greatly improved the purification efficiency of the recombinant ompA–Fc. Therefore, the *P. pastoris* expression system is suitable for expressing the recombinant chicken IgY Fc fusion protein.

Fragment crystallizable domain bears the recognition signal for specific cellular FcR, which serves as an interaction niche for immune effectors. FcR exists on the surface of many congenital immune cells, especially macrophages. The Fc–FcR interactions can activate macrophages and mediate antigen capture; it can also intensively influence the cytokine production of the stimulated macrophages (Amigorena and Bonnerot, 1999). The most distinguished feature of macrophage activation is the increase in phagocytic activity, which facilitates the antigen presentation to T cells for triggering adaptive immune responses (Sun et al., 2015). The linked IgY Fc enhanced the fluorescent antibody staining of ompA phagocytosed by chicken peritoneal macrophages in IFA assay, which implied that the fused Fc largely retained its natural features and mediated the interaction with macrophages. Furthermore, the linked IgY Fc promoted the expression of MHC-II molecules on peritoneal macrophages. MHC-II molecules are highly expressed on specialized APCs, such as B cells, dendritic cells, and macrophages. The upregulation of MHC-II facilitated APC to present peptides, derived from the processing of exogenous antigen, to CD4+ T cells to drive the activation of naïve T cells and elicit help or regulation from CD4 effector or regulatory T cells. Thus, our results imply that the linked chicken IgY Fc is likely to confer the fused antigens more characteristics to accelerate the antigen processing of macrophages, thereby improving specific immune response.

In the present study, we also found that TPPPS, a polysaccharide extracted from Taishan *P. massoniana* pollen, can increase macrophage proliferation and activation. However, the promotion mechanism of macrophage proliferation by TPPPS needs further research. Previous studies suggested that polysaccharide-activated macrophage function could be due to the complex monosaccharide composition of polysaccharides and the mechanism of recognition by macrophages, which include different receptors cooperating with one another to activate redundant signaling pathways (Schepetkin and Quinn, 2006). During activation, macrophages initiate phagocytosis, which is the first step in the macrophage response against pathogens and microbes (Henneke and Golenbock, 2004). Activated macrophages produce chemical signals and cytokines that contribute to the immune responses. In the present study, TPPPS promoted the secretion of NO and TNF-α, which are produced by activated macrophages and serve as the activation signals that indicate the enhancement of macrophage activity. TPPPS as the adjuvant also showed a high performance in improving the immune effect of the fused ompA–Fc, including humoral and cellular immunities. Recent studies have reported that TPPPS is composed of three types of polysaccharides (named TPPPS1–3), and each polysaccharide component is composed of different monosaccharides and exhibits antioxidant, antivirus, and immunomodulating effects, indicating their synergistic effects on facilitating the immune function of organisms (Yang et al., 2015). Considering the findings of this study, we believe that TPPPS has a great application potential in the development of novel subunit vaccines.

## Conclusion

This study demonstrates that the Fc linked onto the antigen can enhance the antigen-processing efficiency of macrophages and the specific immune response induced by the antigen. Moreover, TPPPS, as a novel macrophage stimulator, can remarkably elevate the expression of the activation markers and promote the vitality and activity of macrophages. Meanwhile, TPPPS as the adjuvant presents good immune-enhancing effects on the fused ompA–Fc subunit vaccine. Thus, TPPPS, combined with the linked Fc, synergistically facilitated the systemic immune response induced by antigen subunit. Overall, our findings provided a new perspective to improve the immune effects of subunit vaccines for poultry disease prevention.

## Author Contributions

RZ, KW, and WD designed research. WD, HZ, HH, JZ, LH, AL, LZ, NM, and PY performed research. WD, KW, and RZ analyzed data. WD, KW, and RZ wrote the paper.

## Conflict of Interest Statement

The authors declare that the research was conducted in the absence of any commercial or financial relationships that could be construed as a potential conflict of interest.

## References

[B1] Amigorena S. Bonnerot C. (1999). Fc receptors for IgG and antigen presentation on MHC class I and class II molecules. *Semin. Immunol.* 11 385–390. 10.1006/smim.1999.0196 10625592

[B2] Brumeanu T. Swiggard W. Steinman R. Bona C. Zaghouani H. (1993). Efficient loading of identical viral peptide onto class II molecules by antigenized immunoglobulin and influenza virus. *J. Exp. Med.* 178 1795–1799. 10.1084/jem.178.5.1795 8228825 PMC2191225

[B3] Cereghino J. L. Cregg J. M. (2000). Heterologous protein expression in the methylotrophic yeast *Pichia pastoris*. *Fems. Microbiol. Rev.* 24 45–66. 10.1111/j.1574-6976.2000.tb00532.x 10640598

[B4] Cook J. Barber B. H. (1995). Recombinant antibodies containing an engineered B-cell epitope capable of eliciting conformation-specific antibody responses. *Vaccine* 13 1770–1778. 10.1016/0264-410X(95)00140-V 8701592

[B5] Cui G. Zhong S. Yang S. Zuo X. Liang M. Sun J. (2013). Effects of Taishan *Pinus massoniana* pollen polysaccharide on the subunit vaccine of *Proteus mirabilis* in birds. *Int. J. Biol. Macromol.* 56 94–98. 10.1016/j.ijbiomac.2013.02.006 23403027

[B6] Denac H. Moser C. Tratschin J. D. Hofmann M. A. (1997). An indirect ELISA for the detection of antibodies against porcine reproductive and respiratory syndrome virus using recombinant nucleocapsid protein as antigen. *J. Virol. Methods.* 65 169–181. 10.1016/S0166-0934(97)02186-1 9186940

[B7] Ding A. H. Nathan C. F. Stuehr D. J. (1988). Release of reactive nitrogen intermediates and reactive oxygen intermediates from mouse peritoneal macrophages. Comparison of activating cytokines and evidence for independent production. *J. Immunol.* 141 2407–2412. 3139757

[B8] Gentry-Weeks C. R. Hultsch A.-L. Kelly S. M. Keith J. M. Curtiss R. (1992). Cloning and sequencing of a gene encoding a 21-kilodalton outer membrane protein from *Bordetella avium* and expression of the gene in *Salmonella typhimurium*. *J. Bacteriol.* 174 7729–7742. 1447140 10.1128/jb.174.23.7729-7742.1992 PMC207487

[B9] Harrington A. T. Castellanos J. A. Ziedalski T. M. Clarridge J. E. Cookson B. T. (2009). Isolation of *Bordetella avium* and novel *Bordetella* strain from patients with respiratory disease. *Emerg. Infect. Dis* 15 72–74. 10.3201/eid1501.071677 19116056 PMC2660683

[B10] Henneke P. Golenbock D. T. (2004). Phagocytosis, innate immunity, and host–pathogen specificity. *J. Exp. Med.* 199 1–4. 10.1084/jem.20031256 14707110 PMC1887728

[B11] Hu X. Zhu R. Liu H. Bi J. Li X. (2007). Study on extraction and antigenicity of the outer-membrane-protein of *Bordetella avium*. *Acta Microbiol. Sin.* 47 714–717. 17944379

[B12] Iorio A. Krishnan S. Myrén K. Lethagen S. McCormick N. Karner P. (2015). Factor consumption for prophylaxis and treatment of bleeding: recombinant factor Ix Fc fusion protein compared with conventional recombinant factor Ix. *Value Health* 18:A660 10.1016/j.jval.2015.09.2396

[B13] Jalava K. Hensel A. Szostak M. Resch S. Lubitz W. (2002). Bacterial ghosts as vaccine candidates for veterinary applications. *J. Control. Release* 85 17–25. 10.1016/S0168-3659(02)00267-5 12480307

[B14] Jefferis R. (2009). Glycosylation as a strategy to improve antibody-based therapeutics. *Nat. Rev. Drug Discov.* 8 226–234. 10.1038/nrd2804 19247305

[B15] Jiang J. Wu C. Gao H. Song J. Li H. (2010). Effects of astragalus polysaccharides on immunologic function of erythrocyte in chickens infected with infectious bursa disease virus. *Vaccine* 28 5614–5616. 10.1016/j.vaccine.2010.06.025 20598783

[B16] Konduru K. Bradfute S. B. Jacques J. Manangeeswaran M. Nakamura S. Morshed S. (2011). Ebola virus glycoprotein Fc fusion protein confers protection against lethal challenge in vaccinated mice. *Vaccine* 29 2968–2977. 10.1016/j.vaccine.2011.01.113 21329775 PMC3070761

[B17] Liu L. Yu C. Wang C. Shao M. Yan Z. Jiang X. (2016). Immuno-enhancement of Taishan *Pinus massoniana* pollen polysaccharides on recombinant *Bordetella avium* ompA expressed in *Pichia pastoris*. *Microb. Pathog.* 95 54–61. 10.1016/j.micpath.2016.03.002 26975477

[B18] Lowry S. F. (1993). Cytokine mediators of immunity and inflammation. *Arch. Surg.* 128 1235–1241. 10.1001/archsurg.1993.01420230063010 8239986

[B19] Lu L. Palaniyandi S. Zeng R. Bai Y. Liu X. Wang Y. (2011). A neonatal Fc receptor-targeted mucosal vaccine strategy effectively induces HIV-1 antigen-specific immunity to genital infection. *J. Virol.* 85 10542–10553. 10.1128/JVI.05441-11 21849464 PMC3187492

[B20] Ma X. Guo Z. Wang D. Hu Y. Shen Z. (2010). Effects of sulfated polysaccharides and their prescriptions on immune response of ND vaccine in chicken. *Carbohydr. Polym.* 82 9–13. 10.1016/j.carbpol.2010.04.013

[B21] Mahapatra S. K. Das S. Bhattacharjee S. Gautam N. Majumdar S. Roy S. (2009). In vitro nicotine-induced oxidative stress in mice peritoneal macrophages: a dose-dependent approach. *Mech. Method.* 19 100–108. 10.1080/15376510802255184 19778253

[B22] Mosmann T. (1983). Rapid colorimetric assay for cellular growth and survival: application to proliferation and cytotoxicity assays. *J. Immunol. Methods* 65 55–63. 10.1016/0022-1759(83)90303-4 6606682

[B23] Mwanza M. Kametler L. Bonai A. Rajli V. Kovacs M. Dutton M. F. (2009). The cytotoxic effect of fumonisin B1 and ochratoxin a on human and pig lymphocytes using the Methyl Thiazol Tetrazolium (MTT) assay. *Mycotoxin Res.* 25 233–238. 10.1007/s12550-009-0033-z 23605153

[B24] Neild A. Murata T. Roy C. R. (2005). Processing and major histocompatibility complex class II presentation of *Legionella pneumophila* antigens by infected macrophages. *Infect. Immun.* 73 2336–2343. 10.1128/IAI.73.4.2336-2343.2005 15784579 PMC1087436

[B25] Nimmerjahn F. Ravetch J. V. (2008). Fcγ receptors as regulators of immune responses. *Nat. Rev. Immunol.* 8 34–47. 10.1038/nri2206 18064051

[B26] Peng W. Si W. Yin L. Liu H. Yu S. Liu S. (2011). *Salmonella* enteritidis ghost vaccine induces effective protection against lethal challenge in specific-pathogen-free chicks. *Immunobiology* 216 558–565. 10.1016/j.imbio.2010.10.001 21247655

[B27] Qin M. Tang X. Yin G. Liu X. Suo J. Geru T. (2016). Chicken igy fc expressed by eimeria mitis enhances the immunogenicity of e. mitis. *Parasit. Vectors* 9 1–7. 10.1186/s13071-016-1451-3 27000834 PMC4802925

[B28] Rath T. Baker K. Dumont J. A. Peters R. T. Jiang H. Qiao S.-W. (2015). Fc-fusion proteins and FcRn: structural insights for longer-lasting and more effective therapeutics. *Crit. Rev. Biotechnol.* 35 235–254. 10.3109/07388551.2013.834293 24156398 PMC4876602

[B29] Roopenian D. C. Akilesh S. (2007). FcRn: the neonatal Fc receptor comes of age. *Nat. Rev. Immunol.* 7 715–725. 10.1038/nri2155 17703228

[B30] Saif Y. M. Moorhead P. Whitmoyer R. (1981). Scanning electron microscopy of tracheas from turkey poults infected with *Alcaligenes faecalis*. *Avian Dis.* 25 730–735. 10.2307/1590004 7316906

[B31] Schepetkin I. A. Quinn M. T. (2006). Botanical polysaccharides: macrophage immunomodulation and therapeutic potential. *Int. Immunopharmacol.* 6 317–333. 10.1016/j.intimp.2005.10.005 16428067

[B32] Sun H. Zhang J. Chen F. Chen X. Zhou Z. Wang H. (2015). Activation of RAW264. 7 macrophages by the polysaccharide from the roots of *Actinidia eriantha* and its molecular mechanisms. *Carbohydr. Polym.* 121 388–402. 10.1016/j.carbpol.2014.12.023 25659714

[B33] Sutterwala F. S. Noel G. J. Clynes R. Mosser D. M. (1997). Selective suppression of interleukin-12 induction after macrophage receptor ligation. *J. Exp. Med.* 185 1977–1985. 10.1084/jem.185.11.1977 9166427 PMC2196339

[B34] Tayra J. T. Kameda M. Yasuhara T. Agari T. Kadota T. Wang F. (2013). The neuroprotective and neurorescue effects of carbamylated erythropoietin Fc fusion protein (CEPO-Fc) in a rat model of Parkinson’s disease. *Brain Res.* 1502 55–70. 10.1016/j.brainres.2013.01.042 23380533

[B35] Temple L. M. Miyamoto D. M. Mehta M. Capitini C. M. Von Stetina S. Barnes H. J. (2010). Identification and characterization of two *Bordetella avium* gene products required for hemagglutination. *Infect. Immun.* 78 2370–2376. 10.1128/IAI.00140-10 20351141 PMC2876549

[B36] Torti C. Prosperi M. Motta D. Digiambenedetto S. Maggiolo F. Paraninfo G. (2012). Factors influencing the normalization of CD4+ T-cell count, percentage and CD4+/CD8+ T-cell ratio in HIV-infected patients on long-term suppressive antiretroviral therapy. *Clin. Microbiol. Infec.* 18 449–458. 10.1111/j.1469-0691.2011.03650.x 21919996

[B37] Wei K. Sun Z. Yan Z. Tan Y. Wang H. Zhu X. (2011). Effects of Taishan *Pinus massoniana* pollen polysaccharide on immune response of rabbit haemorrhagic disease tissue inactivated vaccine and on production performance of Rex rabbits. *Vaccine* 29 2530–2536. 10.1016/j.vaccine.2011.01.068 21295100

[B38] Yang S. Wei K. Jia F. Zhao X. Cui G. Guo F. (2015). Characterization and biological activity of Taishan *Pinus massoniana* pollen polysaccharide in vitro. *PLoS ONE* 10:e0115638 10.1371/journal.pone.0115638 PMC4363904 25782009

[B39] Zaharatos G. J. Yu J. Pace C. Song Y. Vasan S. Ho D. D. (2011). HIV-1 and influenza antigens synthetically linked to IgG2a Fc elicit superior humoral responses compared to unmodified antigens in mice. *Vaccine* 30 42–50. 10.1016/j.vaccine.2011.10.05 22064264

